# Spontaneous Coronary Artery Dissection: A Case Report

**Published:** 2015-07-03

**Authors:** Vahid Mokhberi, Babak Bagheri, Seyfollah Navidi, Seyed Mohammad Amini

**Affiliations:** Department of Heart, Faculty of Medicine, Mazandaran University of Medical Sciences, Sari, Iran.

**Keywords:** *Acute coronary syndrome*, *Dissection*, *Death*, *sudden*, *cardiac*, *Coronary artery disease*

## Abstract

Spontaneous coronary artery dissection (SCAD) is a rare and important cause of acute coronary syndrome and sudden cardiac death. Various etiologies are thought to be responsible for this condition, among which underlying atherosclerosis seems to be the most common. SCAD is predominant in women and is usually diagnosed via coronary artery angiography. Therapeutic interventions include medical therapy, percutaneous coronary artery intervention, and surgery based on lesion characteristics. We describe a 36-year-old woman with SCAD presenting with acute chest pain to Fatemeh-Zahra Hospital, Sari, Iran. The patient had no current atherosclerosis risk factors and had given birth 6 months previously. Coronary angiography was performed due to the persistence of the chest pain after initial management, and a spontaneous dissection of the left anterior descending artery was observed. She underwent coronary artery bypass graft and was discharged in good condition.

## Introduction

Spontaneous coronary artery dissection (SCAD) is a rare cause of acute coronary syndrome and sudden cardiac death and occurs mainly in young adults, especially women, who are otherwise healthy. 

Atherosclerosis, peripartum period, inflammatory and connective tissue disorders (Ehlers-Danlos and Marfan syndromes), heavy exercise, and certain drugs (oral contraceptives, cocaine, etc.) are considered predisposing factors for SCAD. The clinical presentation of this complication depends on the flow limiting severity of the coronary artery dissection and ranges from asymptomatic to acute coronary syndromes and ventricular arrhythmias to sudden cardiac death. Diagnosis is mostly made at angiography, and such various therapeutic modalities as medical therapy and interventional and surgical procedures are applied based on the severity and site of the lesion.^1^^-^^4^

***Case Presentation***

A 36-year-old woman presented to the emergency department complaining of spontaneous and severe retrosternal pain, radiating to the left arm and associated with nausea, vomiting, and dyspnea. The patient had no history of hypertension, diabetes, dyslipidemia, smoking, or familial heart disease. Her last delivery was more than 6 months previously.

Physical examination was unremarkable. Blood pressure and heart rate were within the normal range, and blood pressure was not significantly different between the right and left hands.

**Figure1:**
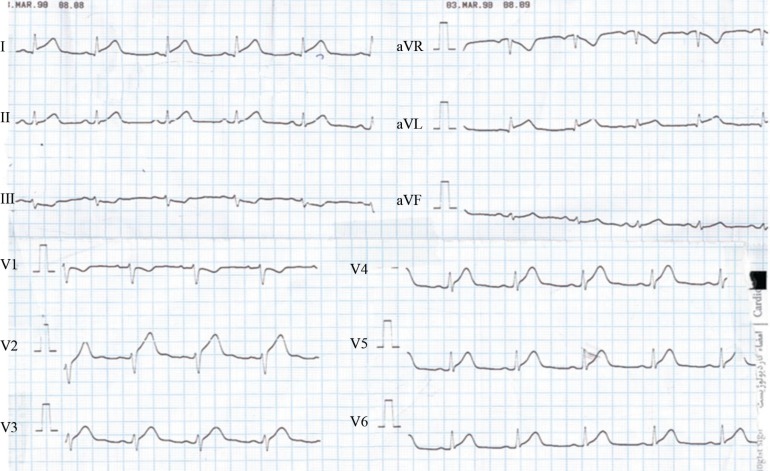
Initial electrocardiogram at presentation, revealing anterolateral ST-segment changes (V_2_-V_6_, I, and aVL leads)

**Figure 2:**
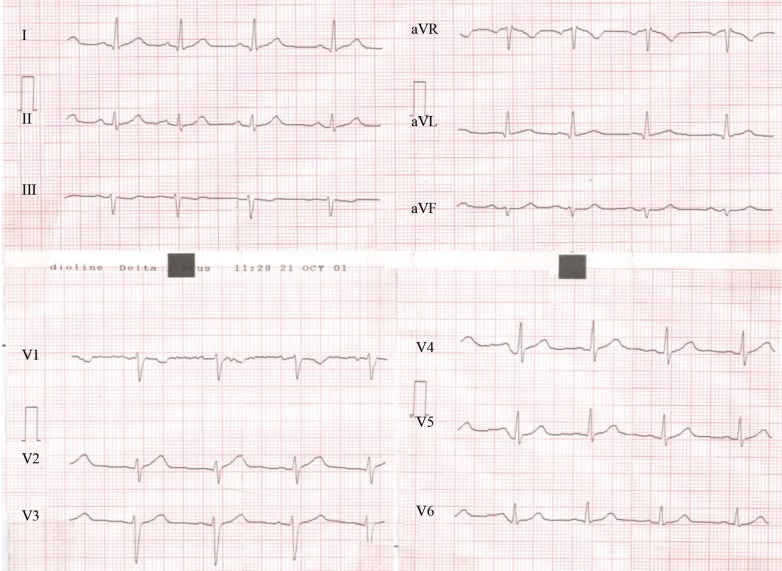
Second electrocardiogram one hour after admission, showing the resolution of the initial changes

The initial electrocardiogram (ECG) at presentation revealed anterolateral ST-segment changes (V_2_-V_6_, I, and aVL leads). The changes were reversed to a large extent in the next ECG, while the pain still persisted (Figures 1 and 2). Laboratory tests were normal except for an elevated serum troponin level. Echocardiography displayed an ejection fraction of 40-45% and hypokinesia of the apex and septal wall of the left ventricle.

Classic treatment for acute coronary syndrome, including aspirin, clopidogrel, metoprolol, atorvastatin, nitroglycerin, and anticoagulant agents, were administered for the patient. Given the ECG dynamic changes, no fibrinolytic agent was applied.

**Figure 3:**
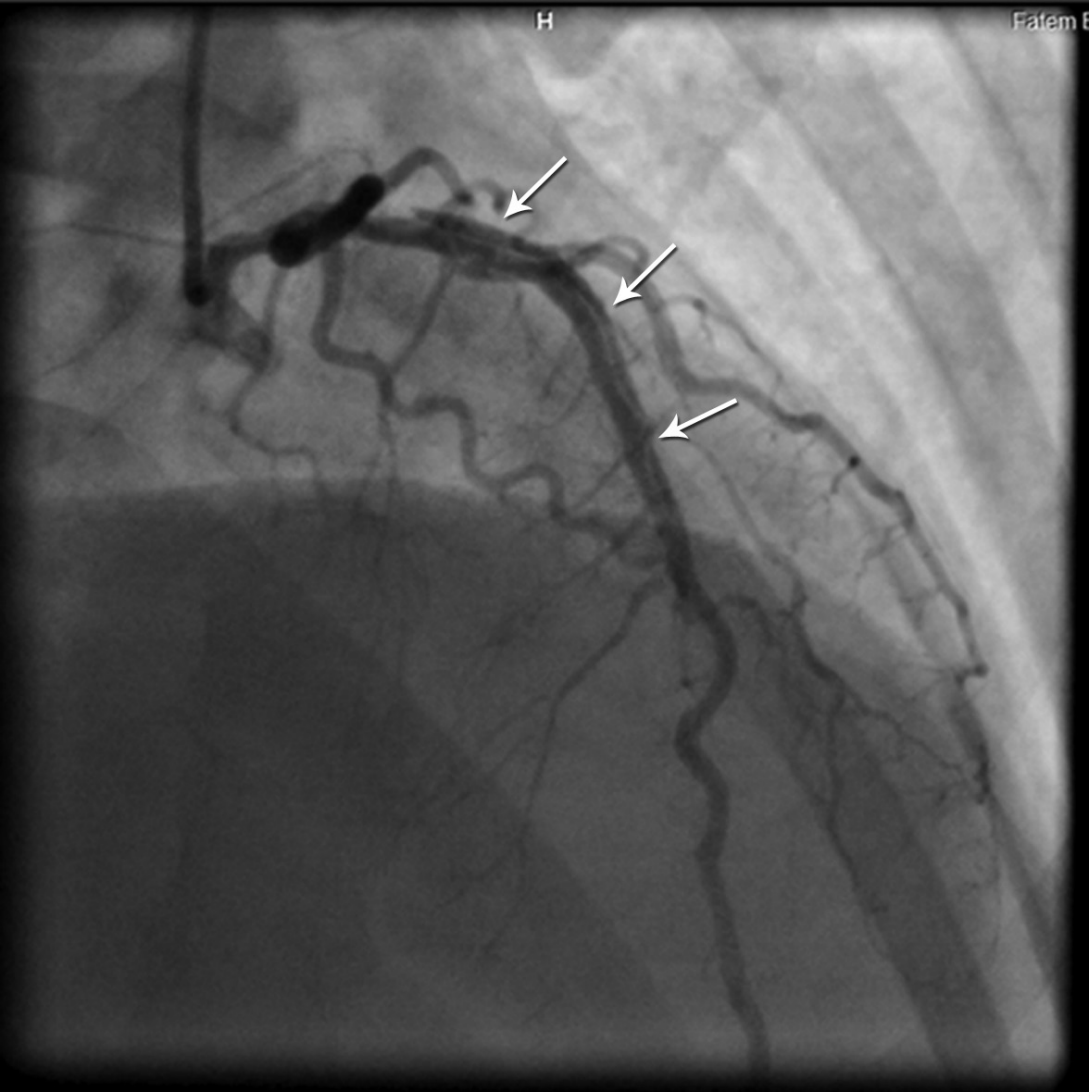
Right anterior oblique coronary angiographic image, showing a long dissection at the proximal portion to the distal part of the left anterior descending artery (arrows)

Coronary angiography was performed due to the persistence of the patient’s chest pain after initial management; it revealed spontaneous dissection of the left anterior descending artery (LAD), involving a great portion of the artery (Figure 3). The patient was transferred to the operating room, where she underwent coronary artery bypass grafting (CABG). She was later discharged home in good condition.

## Discussion

The first case of SCAD was described in 1931 in the autopsy of a 42-year-old woman.^3^ The overall incidence of SCAD is estimated to range from 0.28% to 1.1% based on angiographic assessments. It seems that the actual incidence is higher due to the substantial number of spontaneous dissections which present as sudden death.^1^ Young women account for approximately 70% of the patients (female to male ratio: 2:1), and 30% of such cases are associated with the peripartum period.^4^^, ^^5^ The most frequent site of dissection is the LAD, accounting for 60% of coronary dissections. The right coronary artery is the second most common site (more common in males), followed by the left main artery.^6^ The dissection mostly occurs between the intima and the media but can also occur between the media and the adventitia.^1^ Atherosclerosis and peripartum period are the two most common causes of SCAD. Generally, patients with SCAD are categorized in four groups.^7^ Patients with hereditary connective tissue disorders associated with a defective arterial wall (e.g. the Marfan and Ehlers-Danlos syndromes) constitute one group. Another group comprises patients with underlying atherosclerosis, especially men at an average age of 55 years. (It is believed that vasculitis and atherosclerosis are responsible for a higher incidence rate of SCAD in patients with lupus.)^3^ The third group consists of women in the peripartum period. (Three-fourths of the cases occur within one day to 3 months postpartum. Increased blood flow, shear stress, and fluctuation in estrogen and relaxin blood levels are believed to be responsible for this event.)^8^^, ^^9^ And the last group is composed of patients with idiopathic SCAD. It is worthy of note that SCAD is also reported in association with severe exercise, chest trauma, and consumption of certain drugs such as cocaine, cyclosporine, 5-fluorouracil, oral contraceptives, and fenfluramine.^1^^, ^^9^

SCAD has a wide range of clinical manifestations varying from mild symptoms and stable angina to myocardial infarction and even cardiogenic shock and arrhythmias. Acute coronary syndrome is the predominant presentation. The diagnosis of SCAD should be considered in all young women presenting with acute coronary syndrome, not least in their peripartum period.^10^

Our patient did not have any risk factors or history of ischemic heart disease. Moreover, she did not have delivery in the preceding 6 months. She presented with acute coronary syndrome and had an elevated serum troponin level.

Coronary angiography is the main diagnostic tool. Imaging techniques such as intravascular ultrasound (IVUS) and optical coherence tomography (OCT) demonstrate more details about the morphology and intramural location of the lesions in case of vague angiographic pictures. IVUS is valuable in the follow-up of treatment.^10^^, ^^11^

Aside from some reports and collections of data, the existing literature contains no randomized clinical trial on the management of SCAD due to its low prevalence.^5^ The therapeutic management of SCAD includes medical treatment, percutaneous coronary intervention, and surgery. Patients with extensive dissections resulting in persistent ischemia are usually treated with surgery or percutaneous coronary intervention, while cases with mild involvement can be treated conservatively with medication.^1^^, ^^2^^, ^^6^ Similar to the management of acute coronary syndrome, medications such as anticoagulants, aspirin, clopidogrel, beta blockers, nitrates, and sometimes calcium channel blockers are applied for SCAD management.^1^^, ^^4^ It is deserving of note that although GP2b-3a inhibitors (theoretically enhancing hematoma formation) are often applied for acute coronary syndrome patients before coronary angiography, there is a paucity of information on their utilization in SCAD. Furthermore, administration of fibrinolytic agents is contraindicated.^4^

Kolleret al.^4^ reported recovery in a patient with postpartum SCAD following the use of prednisone and cyclophosphamide in addition to conventional medical therapy. In small lesions requiring intervention, coronary stent implantation can be used after accurate identification of false and true lumens; nevertheless, surgical management is preferred in multi-vessel involvement or large lesions, particularly involving the left main artery.^6^

Our patient underwent coronary angiography, given the sustained ischemia and dynamic changes in her ECGs, and CABG, given the extensive involvement of her LAD (Figure 3).

During the follow-up period, it is necessary to prescribe beta blockers, aspirin, and cholesterol-lowering agents for acute coronary syndrome patients and calcium channel blockers and nitrates in case of coronary artery spasm for the long term. Generally, the prognosis is good in the treatment of SCAD. In patients with a history of peripartum SCAD, the risk of coronary artery dissection is increased in subsequent pregnancies and advanced age. Routine follow-up angiography is not recommended in patients with SCAD, but it is reasonable to perform nuclear perfusion scan during the follow-up of patients with the involvement of the large vessels.^1^

## Conclusion

SCAD is an uncommon disease which occurs in young women. Most patients present with acute coronary syndrome. The early recognition and diagnosis of SCAD is important given the high mortality associated with this condition. The diagnosis of SCAD should be considered in the differential diagnosis of chest pain, especially in younger patients.

## Notes:

***This paper should be cited as: ***Mokhberi V, Bagheri B, Navidi S, Amini SM. Spontaneous Coronary Artery Dissection: A Case Report. J The Univ Heart Ctr 2015;10(3):159-162.
